# Related Risk Factors and Treatment Management of Psoriatic Arthritis Complicated With Cardiovascular Disease

**DOI:** 10.3389/fcvm.2022.835439

**Published:** 2022-04-06

**Authors:** Zhoulan Zheng, Qianyu Guo, Dan Ma, Xuexue Wang, Chengqiang Zhang, Haiyao Wang, Liyun Zhang, Gailian Zhang

**Affiliations:** ^1^Third Hospital of Shanxi Medical University, Shanxi Bethune Hospital, Shanxi Academy of Medical Sciences, Tongji Shanxi Hospital, Taiyuan, China; ^2^The Fifth Clinical Medical College of Shanxi Medical University, Taiyuan, China

**Keywords:** psoriatic arthritis, cardiovascular disease, traditional cardiovascular risk factors, inflammation risk, treatment management

## Abstract

Psoriatic arthritis (PsA) is a chronic autoimmune inflammatory joint disease related to psoriasis (PsO). The risk of PsA patients with cardiovascular disease (CVD) is significantly higher than that of the general population. At present, the relevant mechanism is not clear, chronic inflammation and traditional cardiovascular risk factors are the most important factors for the increased risk of CVD in PsA patients. Early assessment of the risk of PsA patients with CVD, and active control of the disease activity of PsA patients and intervention of traditional cardiovascular risk factors can delay the progression of CVD risk. This article reviews the epidemiology and pathogenesis between PsA and CVD, and reviews the latest developments in the risk assessment and management of CVD in PsA patients.

## Introduction

According to the World Health Organization, 17.7 million people die from cardiovascular disease (CVD) each year, accounting for 31% of all deaths worldwide (1). Psoriatic arthritis (PsA)is a chronic, inflammatory and immune-mediated disease that affects up to 30% of patients with psoriasis (PsO) (2). PsA was once considered a relatively mild disease, but more and more studies have shown that it can bring a huge economic burden to patients and families, especially with CVD, including Ischemic Heart Disease, all stable and unstable angina, Myocardial infarction, transient ischemic attack, coronary artery insufficiency, peripheral artery disease, stroke, congestive heart failure (3). Studies have found that CVD is the most common comorbidity in patients with PsA (4, 5). At present, with the advent of targeted therapy, the joint and skin performance of PsA patients has been greatly improved. However, due to insufficient recognition or treatment of PsA combined with CVD, serious morbidity and even mortality have been caused (6). The EULAR for PsA pharmacological management in 2019 recommends that when dealing with PsA patients, comorbidities such as metabolic syndrome and CVD should be considered (7). This article will review the research progress in the epidemiology, pathogenesis, risk assessment and management of PsA combined with CVD.

## Epidemiology of PsA With CVD

Compared with the general population, the incidence and mortality of CVD in PsA patients have increased (8–11). CVD-related risk factors and morbidity risks in the PsA population are also higher than those in the general population (12, 13). Polachek et al. (14) conducted a meta-analysis and included 11 studies, the results showed that compared with the general population, the incidence of CVD of PsA increased by 43%, and the risk of incidence increased by 55% in 2017. Among them, myocardial infarction, cerebrovascular disease and the risk of heart failure increased by 68%, 22%, and 31%. Schieir et al. (15) stated in another systematic review and meta-analysis that the risk of myocardial infarction in patients with PsA is significantly increased, even after adjustment for traditional cardiovascular risk factors, the risk of PsA combined with CVD is still significantly increased. A large population-based study in 2019 found that the risk of PsA patients with CVD increased by 29%, and also showed that the increase in CVD risk in PsA and PsO was similar (16). However, some studies have found that compared with PsO patients, PsA patients have a higher burden of carotid artery plaque and a higher incidence of combined CVD (17, 18). But compared with RA, AS and diabetes, patients with PsA have a similar CVD risk (19–21). In recent years, studies have shown that CVD is the main cause of death in PsA patients, Juneblad et al. (22) used Swedish national registration data to compare 464 PsA patients with the general population and found that the standard mortality rate of CVD in PsA patients was significantly higher. Clinicians should pay attention to the comorbidities of PsA patients, multidisciplinary cooperation, and reduce mortality.

## Risk Factors of PsA With CVD

### Chronic Inflammation and Autoimmune Factors

**Figure 1:**
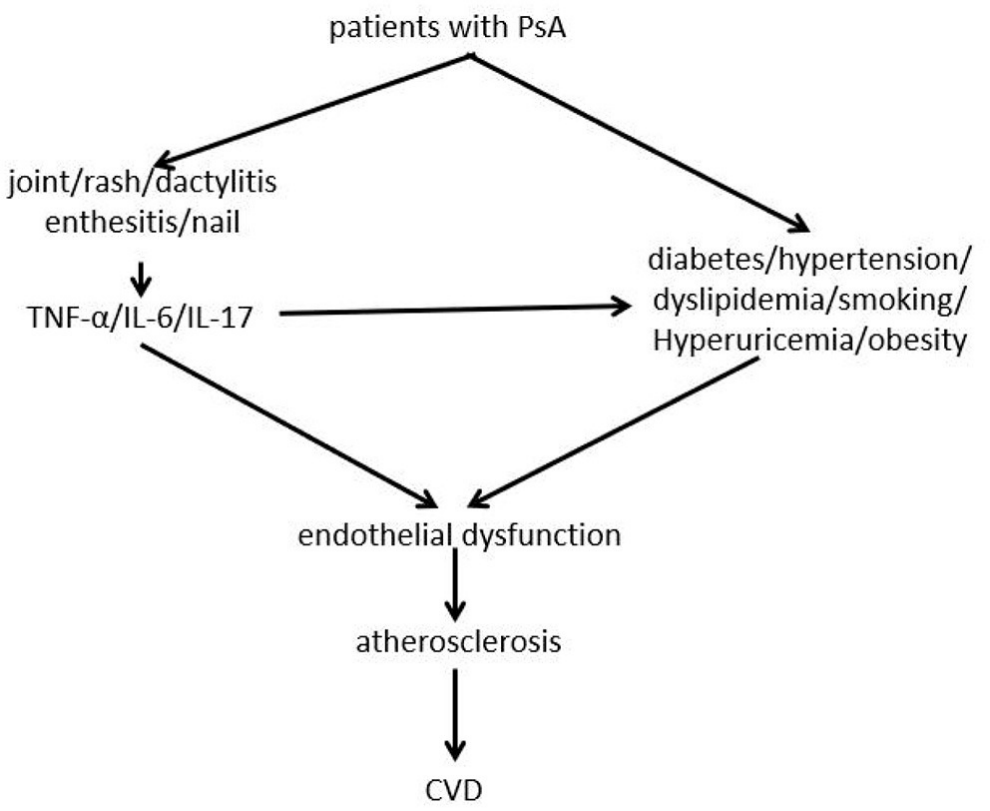
Pathogenesis of psoriatic arthritis complicated with cardiovascular disease.

The increased risk of CVD cannot be fully explained by traditional cardiovascular risk factors, and PsA inflammation is considered to be the main reason for the increased risk of PsA combined with CVD. There is evidence that the pathogenesis of CVD includes systemic inflammation, insulin resistance, dyslipidemia, angiogenesis, oxidative stress, and endothelial dysfunction (23). Studies have shown that many pro-inflammatory cytokines, including TNF, IL-6, and IL-17, are involved in the pathogenesis of PsA, as well as in the pathogenesis of endothelial dysfunction and atherosclerosis (24) (Figure 1). These inflammatory factors suggest that PsA is potentially related to CVD. In addition, systemic inflammation in PsA patients is thought to change lipid structure and function, thereby forming a pro-atherosclerotic profile, this lipid imbalance has been confirmed in PsA cross-sectional studies, although causal data is lacking (25, 26). Systemic inflammation of PsO can lead to insulin resistance, which in turn leads to endothelial dysfunction, atherosclerosis and ultimately CVD (27). In addition to systemic inflammatory factors, chronic recurring inflammation is also involved in the occurrence of PsA-related CVD, because the number and duration of disease activity will increase the risk of CVD. Studies have shown that cumulative inflammation is related to arteriosclerosis in PsA patients, after correcting for traditional cardiovascular risk factors, chronic inflammation still plays an important role in accelerating the development of cardiovascular risk in PsA patients (28–31). The results of a meta-analysis also showed that PsA-related subclinical atherosclerosis and endothelial dysfunction, suggesting that chronic inflammation plays an important role in its pathogenesis, independently and/or in conjunction with traditional cardiovascular risk factors, increase the risk of CVD (32). PsA patients with tendon enthesitis and/or structural damage are at high risk of CVD (33). A cross-sectional study showed that nail involvement in PsA patients is independently related to carotid plaque, and nail involvement is related to severe skin manifestations and joint involvement, which is caused by increased inflammation burden (34). Baseline increases in joint counts, index inflammation counts, and erythrocyte sedimentation rate (ESR) count levels in patients with PsA are independently associated with increases in cardiovascular events (CVE) (35, 36). In short, inflammation is the core mechanism of PsA combined with CVD, however, the data on the effect of PsA-targeted inflammation on cardiometabolism are limited, and further research is needed.

### Traditional Cardiovascular Risk Factors

Studies have found that traditional cardiovascular risk factors (obesity, hypertension, diabetes, dyslipidemia, metabolic syndrome, smoking) are related to PsA, and it also shows that PsA is closely related to increased CVD risk (37). A large study report in 2017 showed that the traditional cardiovascular risk factors of PsA patients are higher than those of RA and PsO (38). In subsequent studies of PsA patients, almost 88% of patients had at least one modifiable cardiovascular risk factor: smoking 17%, type 2 diabetes 13%, hypertension 45%, dyslipidemia 50%, overweight or obesity >75% (39). Compared with those PsA patients without CVD risk factors, PsA patients with more CVD risk factors showed higher disease activity (40). Among them, the obesity factor is more important, and it has been proven to promote the production of IL-17 in adipose tissue and peripheral tissue, and IL-17 is involved in the pathogenesis of obesity and PsA (41). PsA can produce a variety of pro-inflammatory cytokines, which can interfere with metabolic activities and affect adipose tissue and lipid distribution. This can lead to type 2 diabetes, metabolic syndrome, hyperlipidemia, hypertension, and obesity, which can have a profound impact on the management of PsA patients (42). Hyperuricemia appears to be an independent risk factor for PsA (43). for PsA patients without CVD, serum uric acid concentration is associated with subclinical atherosclerosis (44). Appropriate control of hyperuricemia and metabolic diseases can improve the outcome of cardiovascular disease in PsA and play a preventive role (45).

### Genetic Factors

First-degree relatives with a family history of premature cardiovascular event (CVE) (males before the age of 55, females before the age of 65) can significantly increase the risk of CVD in patients with inflammation and the general population (46). At present, there are few genetic research data on PsA patients with CVD, and more data are about the role of genes in the human leukocyte antigen (HLA) region in RA patients with CVD risk. Other genetic polymorphisms in different inflammatory and metabolic pathways located inside and outside the HLA region appear to increase the risk of CVD in RA patients (47, 48).

## Risk Assessment of PsA With CVD

In order to achieve better management and prevention of patients with PsA combined with CVD, it is necessary to carry out relevant assessments of their risks (Table 1). Studies have shown that high ESR values are related to the high burden of atherosclerosis and clinical CVD in PsA patients. Another study on the levels of serum cytokines and adhesion molecules related to endothelial function has found that ESR and DAS28 in endothelin-1 and PsA related, suggesting that the progression of PsA combined with CVD may be slowed by controlling ESR and endothelin-1 levels (35, 78). PsA patients have a moderately increased risk of hyperlipoproteinemia (a) which may help improve the CVD risk assessment of PsA patients (79). Imaging examinations such as ultrasound and coronary CT can help assess the risk of CVD in PsA. Study found that 39% of PsA patients showed carotid plaque formation on ultrasound examination (80). Another meta-analysis showed that in patients with PsA, the common carotid artery intima-media thickness (CCA-IMT) increased, and the blood flow-mediated dilatation (FMD) decreased (32). It is suggested that ultrasound can be used to screen and monitor carotid artery plaque, CCA-IMT and brachial artery FMD to predict the possibility of CVD. However, brachial artery FMD is easily affected by the level of technique, which greatly limits its reproducibility and outcome correlation. CCA-IMT ultrasound technology is safe and reproducible, but the accuracy of different doctors may vary. Current studies have found that CCA-IMT can be used as a surrogate marker for atherosclerosis and CVD risk, the HR for CCA-IMT increased by 0.1 mm and related CVE was 1.65 (81). After carotid ultrasound assessment, patients with PsA are more often reclassified into a very high-scoring risk category than the control group, which is explained independently by disease activity (82–84). A recent multicenter study also found that both PsO and PsA are associated with an increased prevalence of coronary artery calcification (85). Szentpetery et al. (29) used coronary CT angiography to evaluate the relationship between PsA and coronary plaques and found that the prevalence of PsA coronary plaques was 76%, compared with 44% in the control group, the total plaque volume was even larger and mixed plaques have a higher incidence in PsA. Mixed plaques contain thin cap fibrous atherosclerosis, which helps PsA with CVD and poor prognosis. The course of arterial stiffness is highly correlated with the risk of CVD, and the risk of CVD in PsA patients can be assessed by detecting the aortic pulse wave velocity (53, 81). At present, EULAR recommends the routine use of Framingham and SCORE for risk scores to calculate the 10-year risk of CVD events in PsA patients. This risk score can easily underestimate the CVD risk of PsA patients, even after adjusting for the traditional risk factors of PsA patients (51, 86). In PsA patients, in addition to the traditional CV risk score, the presence of higher DAPSA and carotid plaque can independently predict CVD events (87). Therefore, the risk assessment of patients with PsA combined with CVD is particularly important.

**Table 1:** Risk assessment of PsA combined with CVD.

**Assessment method**	**Evaluation metrics**
Scale	DAPSA, MDA, Framingham, SCORE (8–60,64,65)
Serology	ESR, endothelin-1, hyperlipoproteinemia (a) (35,49–50)
Videography	Ultrasound (CCA-IMT, FMD, carotid plaque, aortic pulse wave velocity), CT coronary angiography (29,32,51–52,57–60)

## Management Risk Factors for CVD With PsA

### Management of Traditional Cardiovascular Risk Factors

The risk of CVD in PsA patients is significantly higher than that of the general population, EULAR recommends that all PsA patients undergo a CVD risk assessment at least once every 5 years to screen and identify risk factors for CVD in order to implement risk management and preventive treatment of CVD, at the same time, it is recommended that when using Framingham and SCORE to assess the 10-year risk of CVD events in PsA patients, if the Framingham score is 10% or the SCORE score is 5%, it is recommended that the patient change lifestyle and use lipid-lowering drugs for treatment (51). For overweight or obese PsA patients, ACR/American Psoriasis Foundation recommends weight loss (88). Weight loss intervention can increase the proportion of patients with PsA who reach minimum disease activity (MDA) (89, 90). Once the MDA level is reached, the MDA level should ideally be extended to prevent the progression of carotid atherosclerosis and arteriosclerosis in PsA patients (91, 92). In addition, studies have shown that exercise has a significant beneficial effect on PsA on disease activity and CVD risk (93). Schieir et al. (15) found in a systematic review and meta-analysis that traditional risk factors are more common when PsA is combined with CVD. Therefore, they support a more comprehensive CVD prevention strategy for this population, with the goal of reducing inflammation and enhancing management of traditional CVD risk factors.

### Reasonable Use of NSAIDs Drugs and Glucocorticoids

There is evidence that cyclooxygenase-2 inhibitors (COXIBs) and NSAIDs increase the risk of CVD. A recent meta-analysis showed that non-selective NSAIDs and COXIBs have an adverse effect on the CVD outcome of PsA patients (52). It may be that the use of NSAIDs is related to arterial stiffness in PsA patients, increasing the risk of CVD (53). Another cohort study from the United Kingdom found that the incidence of major adverse cardiovascular events (MACE) in PsA patients receiving glucocorticoid therapy was significantly higher (49). An 11-year retrospective study showed that the increased burden of inflammation reflected by elevated CRP levels in PsA patients was associated with an increased risk of CV events, while the use of NSAIDs in PsA patients significantly reduced the risk of CVD (3). In PsA patients, the use of NSAIDs or glucocorticoid therapy is associated with a high risk of new-onset hypertension. Doctors should pay attention to the early diagnosis of hypertension during treatment with such drugs, reduce the risk of PsA combined with CVD (50). There are few direct data on this relationship in PsA, and further research is needed. EULAR recommends that NSAIDs should be used cautiously in patients with CVD records or CVD risk factors for PsA, because these drugs are usually essential when dealing with the disease activity of PsA patients, clinicians should evaluate them based on the patient's specific conditions. Then use NSAIDs according to specific treatment guidelines, and glucocorticoids can eliminate the harm of inflammation to CVD, but it will also increase the risk of CVD. When treating patients with active PsA, the lowest effective dose can be given short-term continuous treatment (51).

### Control PsA Disease Activity

EULAR proposed that controlling disease activities can reduce the risk of PsA combined with CVD (Table 2). Current studies have shown that the use of biologics in PsA patients is related to the reduction of major CVD (57, 72). And proper disease activity control will reduce the use of non-steroidal anti-inflammatory drugs and glucocorticoids, which will ultimately help reduce the incidence of new CVE in these patients (35, 94). The most common first-line csDMARDs for the treatment of PsA patients is methotrexate (MTX) (70.9%), and the most common first-line bsDMARDs is adalimumab (30.8%) (95). There is conflicting evidence regarding the clinical cardiovascular end points of myocardial infarction, stroke, and cardiovascular-related death in PsA patients treated with biologics. A meta-analysis of 10 cohort studies including patients with RA, PsO, and PsA found that MTX treatment can reduce overall cardiovascular risk by 21% and myocardial infarction risk by 18%. MTX may be reduce cardiovascular risk by suppressing inflammation (54). Another study found that the vascular endothelial function of PsA patients in the MTX group improved more than that in the TNF-αi-MTX group (55). A recent meta-analysis of observational studies of 14 RA patients found that compared with TNF-αi treatment, methotrexate has an increased risk of MACE and stroke (56). Eder et al. (35) investigated the incidence of CVE in a large PsA clinic and found that there was no difference in MACE between TNF-αi, MTX, and untreated PsA patients. Studies have found that TNF-αi inhibitors can delay the progression of subclinical atherosclerosis, reduce arterial stiffness, and reduce the risk of CVD while treating PsA inflammation (57–64). The reduced cardiovascular morbidity observed in PsA patients receiving TNF-αi therapy may be partly due to its beneficial effects on complement (65). Another meta-analysis showed that the use of TNF-αi to target systemic inflammation can provide cardioprotection for patients with PsO and/or PsA, and can reduce the risk of CVD (66). A systematic review and meta-analysis by Roubille et al. (52). In 2015 showed that in patients with PsO and PsA, biologics and other DMARDs may be related to reducing the risk of CVD, but compared with RA, the evidence is not conclusive. Data from two large commercial databases in the United States show that in patients with PsO or PsA, compared with TNF-αi, there is no difference in the MACE or mortality of uzumumab (IL-12/23) initiation of treatment (67). A national cohort study in 2021 showed that compared with TNF-αi, patients with PsA using IL-12/23 and IL-17 are at greater MACE risk (68). Studies have found that Apremilast can help restore vascular endothelial dysfunction and stability, prevent the progression of atherosclerotic plaque, thereby reducing the risk of CVD (69–71), this is in contrast to a recent study by Ferguson et al. (73). Studies have found that compared with TNF-αi, there is no difference in the risk of MACE for major adverse cardiovascular events in PsA patients using Apremilast (68, 72). Tofacitinib treatment of PsA patients increases the risk of dyslipidemia, while the risk of CVD decreases (74–76). But a real-world study found that tofacitinib has a higher incidence of MACE when treating PsA patients (77). Regarding the effect of drugs on the incidence and mortality of CVD in the PsA population, large-scale, prospective, adequately controlled and powerful studies are still needed.

**Table 2:** Risk of CVD in patients with PsA after drug treatment.

**Drug**	**Favorable evidence for CVD**	**Unfavorable evidence for CVD**
Glucocorticd		1. The incidence of MACE in PsA patients receiving glucocorticoid therapy is significantly higher (49) 2. Glucocorticoid therapy increases the risk of hypertension in PsA patients, leading to an increased risk of CVD (50) 3. Glucocorticoids can eliminate the harm of PsA inflammation to CVD, but it can also increase the risk of CVD (51)
NSAIDs	1. the use of NSAIDs in PsA patients can improve inflammation and significantly reduce the risk of CVD (3)	1. Non-selective NSAIDs and COXIBs have adverse effects on the CVD outcome of PsA patients (52) 2. The use of NSAIDs is associated with arterial stiffness in PsA patients and increases the risk of CVD (53) 3. Treatment of NSAIDs in PsA patients increases the risk of hypertension, leading to an increased risk of CVD (50)
csDMARDs	1. Methotrexate may reduce cardiovascular risk by suppressing inflammation (54) 2. Vascular endothelial function of PsA patients in the MTX group improved more than that in the TNF-αi-MTX group (55) 3. There is no difference in MACE between TNF-αi, MTX and untreated PsA patients (35)	1. Compared with TNF-αi treatment of RA, methotrexate has an increased risk of MACE and stroke (56)
bsDMARDs-TNF-αi	1. TNF-αi inhibitors can delay the progression of subclinical atherosclerosis, reduce arterial stiffness, and reduce the risk of CVD while treating PsA inflammation (57–64) 2. The reduced cardiovascular morbidity observed in PsA patients receiving TNF-αi therapy may be partly due to its beneficial effects on complement (65) 3. TNF-αi targeting systemic inflammation can provide cardioprotection for patients with PsO and/or PsA (66)	
bsDMARDs-IL-17/ IL-12/23	1. In patients with PsO or PsA, compared with TNF-αi, there was no difference in the MACE or mortality of ulinumumab (IL-12/23) initiation of treatment (67)	1. Compared with TNF-αi, patients with PsA using IL-12/23 and IL-17 have a greater MACE risk (68)
tsDMARDs-PDE-4	1. Apremilast can help restore vascular endothelial dysfunction and stability, prevent the progression of atherosclerotic plaque, thereby reducing the risk of CVD (69–71) 2. Compared with TNF-αi, there is no difference in the risk of MACE for major adverse cardiovascular events in PsA patients using Apremilast (68, 72)	1. Apremilast can improve the disease activity of patients with PsO, but cannot improve vascular endothelial function (73)
tsDMARDs-JAKi	1. Tofacitinib treatment of PsA patients increases the risk of dyslipidemia, while the risk of CVE decreases (74–76)	1. A real-world study found that tofacitinib has a higher incidence of MACE when treating PsA patients (77)

## Conclusion

With the improvement of the level of diagnosis and treatment, more and more evidences show that the CVD burden of PsA patients has increased significantly. In terms of management, it is recommended to intervene in the traditional risk factors of CVD that are increased in PsA, and then further research is needed on the impact of anti-inflammatory treatments, especially DMARDs and biological agents (including TNF-αi inhibitors) on the burden of CVD. In addition to considering traditional risk factors, chronic and systemic inflammation may lead to the accelerated development of atherosclerosis, which can be measured by some non-invasive techniques, early identification, early treatment, and reduced incidence rate and death rate.

## Author Contributions

ZZ: literature search and writing. QG and DM: suggestions and revision points. XW and HW: literature search. CZ and LZ: propose. GZ: provide ideas and suggestions. All authors contributed to the article and approved the submitted version.

## Funding

This work was supported in part by the Shanxi Province Applied Basic Research Project (201801D121201) and the Shanxi Province Overseas Students Science and Technology Activities Selection Funding Project (20210003).

## Conflict of Interest

The authors declare that the research was conducted in the absence of any commercial or financial relationships that could be construed as a potential conflict of interest.

## Publisher's Note

All claims expressed in this article are solely those of the authors and do not necessarily represent those of their affiliated organizations, or those of the publisher, the editors and the reviewers. Any product that may be evaluated in this article, or claim that may be made by its manufacturer, is not guaranteed or endorsed by the publisher.
